# Concomitant presentation of carpal tunnel syndrome and trigger finger

**DOI:** 10.1186/1749-7221-4-13

**Published:** 2009-08-25

**Authors:** Stephen A Rottgers, Davis Lewis, Ronit A Wollstein

**Affiliations:** 1 Department of Surgery, Division of Plastic and Reconstructive Surgery, University of Pittsburgh Medical Center, Pittsburgh, PA, USA; 2 Department of Orthopedic Surgery, University of Pittsburgh Medical Center, Pittsburgh, PA, USA; 3 Department of Surgery, Veterans Affairs Medical Center, Pittsburgh, PA, USA

## Abstract

**Background:**

Carpal tunnel syndrome (CTS) and trigger finger (TF) are common conditions that may occur in the same patient. The etiology of most cases is unknown. The purpose of this study was to evaluate the rate of concomitant occurrence of these two conditions at presentation and to compare the concomitant occurrence in normal and diabetic patients.

**Methods:**

One-hundred and eight consecutive subjects presenting to our hand clinic with CTS and/or TF were evaluated. The existence of both of these conditions was documented through a standard history and physical examination. The definition of trigger finger was determined by tenderness over the A1 pulley, catching, clicking or locking. CTS was defined in the presence of at least two of the following: numbness and tingling in a median nerve distribution, motor and sensory nerve loss (median nerve), a positive Tinel's or Phalen's test and positive electrophysiologic studies.

**Results:**

The average age of the participants was 62.2 ± 13.6 years. Sixty-seven patients presented with symptoms and signs of CTS (62%), 41 (38%) subjects with signs and symptoms of TF. Following further evaluation, 66 patients (61%) had evidence of concomitant CTS and TF. Fifty-seven patients (53% of all study patients) had diabetes. The rate of subjects with diabetes was similar among the groups (p = 0.8, Chi-square test).

**Conclusion:**

CTS and TF commonly occur together at presentation though the symptoms of one condition will be more prominent. Our results support a common local mechanism that may be unrelated to the presence of diabetes. We recommend evaluation for both conditions at the time of presentation.

## Introduction

Trigger finger (TF) or stenosing tenosynovitis and carpal tunnel syndrome (CTS) together are in all probability the most common conditions treated by the hand surgeon. Previous studies have suggested a significant concurrence rate between these conditions, but the implications of this have not been fully explored [1-3].

Some studies hypothesize that surgery for one entity will cause the other or that the carpal tunnel syndrome that is associated with the appearance of trigger digits is a separate entity [2,4]. We suggest these two conditions often coexist at presentation.

The goal of this study was to quantify the co-existence of these two entities in patients at presentation and to compare this coexistence between patients with and without diabetes. The etiology of both TF and CTS is unknown. Anatomically, they both occur at potential constriction points in the hand and wrist. It is possible that there is a common etiology present in many cases, and the relationship to diabetes may shed light on this common etiology.

## Patients and Methods

All consecutive patients presenting to the hand clinic between June 1, 2007 and January 31, 2008 with complaints attributed to CTS or TF were included in the study. Patients in whom the diagnosis was not clear or had no electrophysiological studies were excluded from the study. The diagnosis was made on the basis of a standard history and physical exam performed by the authors. All CTS patients had nerve conduction tests (NCTs) and electromyography (EMG) consistent with CTS. Approval for this prospective observational study was obtained from the Veterans Administration of Pittsburgh Health System IRB.

Cases of TF were defined by a history and/or presence of catching, clicking or locking and tenderness over the A1 pulley. CTS was defined by the presence of at least two of the following: numbness or tingling in the median nerve distribution, diminished median nerve motor function, or a positive Tinel's or Phalen's test as well as the typical history of nighttime pain or numbness and tingling, symptoms while driving, overhead work, holding the phone for prolonged periods of time. Patient demographics were obtained from the electronic medical record and were catalogued along with pertinent historical/physical findings.

Data included the primary complaint (trigger finger versus CTS), presence/absence of the other condition, digits involved, dominant hand, affected hand, associated hand pathologies, previous hand surgeries, any systemic diseases, presence of diabetes and the presence of Dupuytren's contracture. Documentation also included the following historical/physical findings: nighttime pain, presence of a painful tendon nodule, clicking with digit extension, digit "sticking" in flexion, grip strength (administered using the Jamar dynamometer and scored using the mean of 3 trials) [5]. visual analogue pain scale score, proximal interphalangeal joint (PIP) contracture (measured using a goniometer on both volar and dorsal aspects), Tinel's sign, Phalen's sign, the presence of thenar atrophy, and abductor pollicis brevis strength measured on a 5 pt scale (0–5) using the manual muscle test as described by the medical research council [6].

Prevalence of carpal tunnel syndrome and trigger finger within the study population was found. Additionally, the percentage of patients with concurrent CTS and trigger finger was calculated. The prevalence of CTS in patients presenting with trigger finger, and trigger finger in patient complaining of CTS were determined. Patients presenting with trigger finger after carpal tunnel release (CTR) and carpal tunnel syndrome following TF release were identified. The incidences of diabetes and disease affecting the dominant hand were compared between study groups (CTS alone, TF alone, and both) using Chi-square statistical tests. The frequency of concurrent CTS and TF was compared between subjects with and without diabetes using the same statistical test. Nighttime waking-up was compared using the Wilcoxon sum of ranks test.

## Results

One hundred and eight consecutive patients presenting to the Veterans Administration (VA) clinic were reviewed. The average age of the participants was 62.2 ± 13.6 years. Patients did not have previous treatment unless specified. All subjects were referred through a neurologist or general practitioner. Ninety-four percent of the subjects were right hand dominant and 59% presented with complaints in their dominant hand. No group (carpal tunnel syndrome, trigger finger, or carpal tunnel syndrome with trigger finger) was more likely to have the dominant hand affected (p = 0.7, Chi square test).

Sixty-seven (62%) of the patients presented with a chief complaint consistent with CTS. Forty-one (38%) patients presented primarily with symptoms of trigger finger. Thirty percent had more than one finger involved on presentation. The ring finger was involved most often (24%) followed by the middle (23%) and little (18%), the index (17%) and thumb (17%). After a focused history and physical exam, ninety (83%) patients were found to have active carpal tunnel syndrome or a history of the condition. Eighty-three (77%) patients had symptoms or history of trigger digit. Concurrent CTS and triggering were found in 66 (61%) of patients on initial presentation.

Fourteen patients had a history of carpal tunnel release prior to their current presentation. Of these, 12 had signs or symptoms consistent with trigger digits, but only 5 (42%) developed the TF symptoms in the same hand where they had undergone previous carpal tunnel surgery. Similarly, of the 12 patients with previous trigger finger releases, 5 had signs of CTS. Only 3 (60%) were ipsilateral to the previous trigger finger.

The most common presenting symptom in patients with carpal tunnel syndrome alone was numbness/tingling in the median nerve distribution, and tenderness over the A1 pulley was the most common presenting sign in trigger finger (Table 1). Twenty-nine percent of patients with TF only woke up at night while 64% of patients with CTS described nighttime symptoms. Of the patients with CTS that woke up, the average was 5.9/7 nights a week because of pain or numbness in the affected hand. Of the TF patients that woke up at night, the average was 3.8/7 nights (P = 0.5). The group with concomitant CTS and TF woke up an average of 4.4/7 nights a week.

**Table 1:** Distribution of the signs and symptoms of TF ad CTS in our population

Symptom	Trigger finger alone%	CTS alone%	Trigger finger and CTS (%)
Painful nodule	82%	0%	62%

Catching	71%	0%	44%

Popping/clicking	47%	0%	36%

Stuck in flexion	12%	0%	8%

Pain over A1 pulley	100%	4%	91%

Decreased grip strength	59%	92%	91%

Median nerve numbness/tingling	6%	100%	80%

Nighttime waking	29%	84%	77%

Tinnel test positive	6%	96%	70%

Phalen test positive	6%	68%	58%

Thenar Atrophy	0%	24%	24%

PIP joint contracture	24%	29%	48%

Thenar Strength/5	4.4	4.0	4.3

Fifty-seven (53%) of the study patients suffered from diabetes. There were 48 patients with diabetes along with CTS and 45 with diabetes and TF. Of the patients with concurrent carpal tunnel syndrome and trigger digit, 36 had type 2 diabetes and 30 did not (p = 0.9, Chi square test). The diabetic population did not differ from the general population demographically. Looking at other systemic diseases, six subjects had a history of hypothyroidism, 4 of these presented with TF. Five subjects had a history of inflammatory arthritis (specifically rheumatoid arthritis), 3 of them presented with CTS. Twenty patients (18.5%) had evidence of Dupuytren's disease at the time of presentation though no significant difference was found between the TF and CTS groups. All but one patient had only Dupuytren's nodules with no contracture. One patient had a contracture of the metacarpophalangeal (MP) joint of 30 degrees.

## Discussion

Our population presented in a manner consistent with the literature. The most common presenting symptom in this study was sensory: numbness and tingling in a median nerve distribution. In their series, Tay et al. found sensory symptoms as the most common presenting symptoms in CTS [7]. However, we also found the number of nights a patient wakes up to be significantly higher for CTS (average 6.9/7 nights a week). This phenomenon has been well documented, but not quantified or compared to TF. Szabo et al. found night pain to be a sensitive symptom predictor (96%) of CTS [8]. Lehtinen et al. found that patients with CTS suffer from fragmentary sleep but found no median nerve impairment when they did wake up at night. They found that surgery significantly reduced the number of nocturnal movements [9-12].

The most common presenting sign in TF was tenderness over the A1 pulley. The incidence of this sign has not been documented in the literature though some studies support a treatment plan based on signs in TF and their duration (thus including tenderness over the A1 pulley) as well as the existence of background disease [13-16]. We found trigger finger most commonly in the ring and not in the thumb as described in the literature [17-19].

Garti et al. evaluated 62 consecutive patients with TF and no signs or symptoms of CTS. They found that nerve conduction studies (NCS) of the median nerve in 39/62 patients had increased distal motor latency [17]. Though they looked for evidence of CTS on NCTs, their numbers (63%) are very similar to the concomitance rate that we found in our study. Kumar et al. evaluated the concomitant clinical presentation of TF and CTS in patients with no background disease and found that 43% of the patients presenting with TF also had CTS [1].

Various authors have commented on the concurrence rate between CTS and TF. These studies have focused on the rate of trigger finger within populations of CTS patients, and on the rate of subsequent trigger finger release following carpal tunnel surgery [4,20]. These studies have hinted that a common pathologic process may underlie these two entities, or that treatment of CTS may predispose patients to subsequent triggering. The concurrence rate of 62% at presentation in this observational study emphasizes the need to evaluate the patient for both conditions at the time of presentation as well as possible preparation of the patient for the likelihood of treatment for both conditions. The fact that both conditions exist at the time of presentation supports the hypothesis of a common etiology over one condition or its treatment as a source of the other. The finding of PIP joint contractures was significantly high when both conditions presented concomitantly. Perhaps this hints at a specific pathology that is more common in concomitant occurrence such as swelling or inflammation, which would also cause a higher rate of PIP joint contracture. This can only be speculated on based on these findings.

We did not find that diabetes predisposed to the concomitant occurrence of the two conditions. This does not support diabetes as the common pathological factor for both conditions but rather supports a local, possibly mechanical etiology for concurrence rather than a systemic cause. The fact that both conditions appear equally in both extremities at least supports the theory that whatever the pathology local or systemic, it affects both hands. We believe an effort to decipher the common pathway for both conditions may be helpful not only in understanding their etiology but also in the management of both.

## Conclusion

CTS and TF commonly occur together at presentation though the symptoms of one condition will be more prominent. Our results support a common local mechanism that may be unrelated to the presence of diabetes. We recommend evaluation for both conditions at the time of presentation.

## Competing interests

The authors declare that they have no competing interests.

## Authors' contributions

SAR drafted the manuscript and analyzed the data. DL collected the data. RW conceived of the study, collected and arranged the data, drafted the manuscript. All authors read and approved the final manuscript.

## References

[B1] Kumar P Chakrabarti I Idiopathic carpal tunnel syndrome and trigger finger: is there an association? J Hand Surg Eur Vol 2009 4 58 59 10.1177/1753193408096015 18936127

[B2] Harada K Nakashima H Teramoto K Nagai T Hoshino Se Yonemitsu H Trigger digits-associated carpal tunnel syndrome: relationship between carpal tunnel release and trigger digits Hand Surg 2005 4 205 208 10.1142/S0218810405002905 16568515

[B3] Hombal JW Owen R Carpal tunnel decompression and trigger digits Hand 1970 4 192 196 10.1016/0072-968X(70)90022-7 5520745

[B4] Hayashi M Uchiyama S Toriumi H Nakagawa H Kamimura M Miyasaka T Carpal tunnel syndrome and development of trigger digit J Clin Neurosci 2005 4 39 41 10.1016/j.jocn.2004.08.005 15639409

[B5] Mathiowetz V Kashman N Volland G Weber K Dowe M Rogers S Grip and pinch strength: normative data for adults Arch Phys Med Rehabil 1985 4 69 74 3970660

[B6] Brandsma JW Schreuders TA Birke JA Piefer A Oostendorp R Manual muscle strength testing: intraobserver and interobserver reliabilities for the intrinsic muscles of the hand J Hand Ther 1995 4 185 190 8535479 10.1016/s0894-1130(12)80014-7

[B7] Tay LB Urkude R Verma KK Clinical profile, electrodiagnosis and outcome in patients with carpal tunnel syndrome: a Singapore perspective Singapore Med J 2006 4 1049 1052 17139401

[B8] Szabo RM Slater RR Jr Farver TB Stanton DB Sharman WK The value of diagnostic testing in carpal tunnel syndrome J Hand Surg [Am] 1999 4 704 714 10.1053/jhsu.1999.0704 10447161

[B9] Gainer JV Jr Nugent GR Carpal tunnel syndrome: report of 430 operations South Med J 1977 4 325 328 847486 10.1097/00007611-197703000-00021

[B10] de Krom MC Knipschild PG Kester AD Spaans F Efficacy of provocative tests for diagnosis of carpal tunnel syndrome Lancet 1990 4 393 395 10.1016/0140-6736(90)90218-T 1968125

[B11] Bartova V Zima T Diagnosis and treatment of carpal tunnel syndrome Ren Fail 1993 4 533 537 10.3109/08860229309054970 8210567

[B12] Lehtinen I Kirjavainen T Hurme M Lauerma H Martikainen K Rauhala E Sleep-related disorders in carpal tunnel syndrome Acta Neurol Scand 1996 4 360 365 8800348 10.1111/j.1600-0404.1996.tb00010.x

[B13] Saldana MJ Trigger digits: diagnosis and treatment J Am Acad Orthop Surg 2001 4 246 252 11476534 10.5435/00124635-200107000-00004

[B14] Patel MR Bassini L Trigger fingers and thumb: when to splint, inject, or operate J Hand Surg [Am] 1992 4 110 113 10.1016/0363-5023(92)90124-8 1538090

[B15] Nimigan AS Ross DC Gan BS Steroid injections in the management of trigger fingers Am J Phys Med Rehabil 2006 4 36 43 10.1097/01.phm.0000184236.81774.b5 16357547

[B16] Rozental TD Zurakowski D Blazar PE Trigger finger: prognostic indicators of recurrence following corticosteroid injection J Bone Joint Surg Am 2008 4 1665 1672 10.2106/JBJS.G.00693 18676896

[B17] Fahey JJ Bollinger JA Trigger-finger in adults and children J Bone Joint Surg Am 1954 4 1200 1218 13211713

[B18] Weilby A Trigger finger. Incidence in children and adults and the possibility of a predisposition in certain age groups Acta Orthop Scand 1970 4 419 427 5502406 10.3109/17453677008991529

[B19] Garti A Velan GJ Moshe W Hendel D ncreased median nerve latency at the carpal tunnel of patients with "trigger finger": comparison of 62 patients and 13 controls Acta Orthop Scand 2001 4 I279 281 10.1080/00016470152846628 11480605

[B20] Assmus H [Tendovaginitis stenosans: a frequent complication of carpal tunnel syndrome] Nervenarzt 2000 4 474 476 10.1007/s001150050609 10919142

